# Extension of the Medial Approach to the Tibial Plateau via an Osteotomy of the Tibial Insertion of the Superficial Medial Collateral Ligament

**DOI:** 10.3390/jcm12165208

**Published:** 2023-08-10

**Authors:** Elmar Herbst, Moritz A. Wessolowski, Michael J. Raschke

**Affiliations:** Department of Trauma, Hand and Reconstructive Surgery, University of Muenster, 48149 Muenster, Germany

**Keywords:** tibial plateau fracture, extended anteromedial approach, distal osteotomy of the superficial medial collateral ligament, epicondyle osteotomy, articular surface

## Abstract

The treatment of medial tibial plateau fractures can be challenging due to poor exposure of the articular surface. Therefore, a medial epicondyle osteotomy may be needed. Current methods describe osteotomy of the medial femoral epicondyle. However, this method requires additional detachment of the medial meniscus in order to ensure proper visualization. The aim of this study is to present a new technique using distal osteotomy of the superficial medial collateral ligament and to analyze the area of the exposed articular surface area. On each of eight fresh-frozen human cadaveric knees (mean age: 79.4 ± 9.4 years), an osteotomy and proximal reflection of the distal insertion of the superficial medial collateral ligament combined with a submeniscal arthrotomy was performed, followed by a medial epicondyle osteotomy. Using a three-dimensional measurement arm (Absolute Arm 8320-7, Hexagon Metrology GmbH), the exposed area was analyzed and compared to the entire medial articular surface using ANOVA (*p* < 0.05). Through the medial epicondyle osteotomy, 39.9 ± 9.7% of the anteromedial articular surface was seen. This area was significantly smaller compared to the osteotomy of the distal insertion of the superficial collateral ligament with an exposed articular surface of 77.2 ± 16.9% (*p* = 0.004). Thus, the distal osteotomy exposed 37.3% more of the articular surface compared to the medial epicondyle osteotomy. None of these techniques were able to adequately expose the posteromedial- and medial-most aspects of the tibial plateau. A distal superficial collateral ligament osteotomy may be superior to a medial epicondyle osteotomy when an extension of the anteromedial approach to the tibial plateau is required. A distal superficial medial collateral ligament osteotomy combines the advantages of better exposure of the medial articular surface with preservation of the blood supply to the medial meniscus. However, surgeons should carefully consider whether such an extended approach is necessary, as it significantly increases invasiveness.

## 1. Introduction

The incidence of tibial plateau fractures is steadily increasing, with 23 cases occurring per 100,000 persons a year, most of which are treated surgically [1,2,3]. In general, tibial plateau fractures occur during high-energy injury mechanisms with axial compression and combined rotation in the coronal and/or axial plane [4]. However, it has been shown that the number of low-energy tibia plateau fractures in elderly patients with osteoporotic bones has increased significantly over the last decade [1,2,5].

With the aforementioned demographic changes, the injury pattern of tibial plateau fractures may also change due to the altered bone quality. Most commonly, a large medial or posteromedial fragment is associated with split depressions in the lateral and posterolateral aspects [4,6]. However, an increasing number of articles describe anteromedial and medial impaction or comminution zones in bicondylar tibial plateau fractures due to a hyperextension and/or varus injury mechanism, observed in about 8–10% of cases [7,8]. Such medial-sided impression fractures are associated with inferior functional outcomes as compared to non-hyperextension injuries [9]. The reasons for inferior outcomes following tibial plateau fractures are often non-anatomic reduction with a residual abnormal medial proximal tibia angle and intra-articular gaps and steps of more than 2–2.5 mm [10]. Such intra-articular steps of more than 2 mm are significantly associated with increased tibiofemoral contact pressure, increasing the risk for post-traumatic osteoarthritis [11,12]. Thus, to reduce the increased risk of non-anatomic fracture reduction and therefore post-traumatic osteoarthritis, adequate intraoperative visualization and a reduction in the fracture are of utmost importance as fluoroscopy alone might not be sufficient [13]. However, with regard to the medial tibial plateau, only the anteromedial third can be visualized with an anteromedial approach due to the tight medial compartment and the broad shape of the superficial medial collateral ligament [14].

A proven option to better visualize the medial tibial plateau is an extension of the anteromedial approach via a medial epicondylar osteotomy. By adopting this approach, 62.3% of the medial tibial articular surface can be directly seen, as shown in a cadaveric study [14]. However, the close anatomic relationship between the superficial and deep medial collateral ligaments and the medial meniscus is a limiting factor, even with a medial epicondylar osteotomy to properly visualize the entire medial articular surface [14,15]. In fact, complete separation of the medial collateral ligaments and the medial meniscus might be necessary to actually expose the medial articular surface.

Thus, the purpose of this study was to describe the extension of an anteromedial approach to the tibial plateau with osteotomy of the tibial insertion of the superficial medial collateral ligament with submeniscal arthrotomy, and to analyze the visible medial tibial articular surface, with this technique in comparison to a medial epicondylar osteotomy. By doing so, the meniscocapsular unit, and therefore, the vascularization of the medial meniscus would stay intact. It was hypothesized that a distal osteotomy of the superficial medial collateral ligament would better expose the medial articular surface of the tibial plateau than a medial epicondylar osteotomy.

## 2. Materials and Methods

For this study, eight unpaired fresh-frozen human cadaveric knees from patients with a mean age of 79.4 ± 9.4 years (range: 65–89) and a BMI of 22.9 ± 5.6 kg/m^2^ (range: 15.5–31.5), and with no history or evidence of knee injury or deformity, were used. All specimens were male to rule out any gender-related size discrepancies. As the study is descriptive in nature, with the primary aim of describing a new extension of the medial approach to the tibial plateau, a number of 8 specimens was considered sufficient. The specimens were obtained from MedCure Inc. (Portland, OR, USA) and dissected following IRB approval (2021-543-f-S).

**Figure 1:**
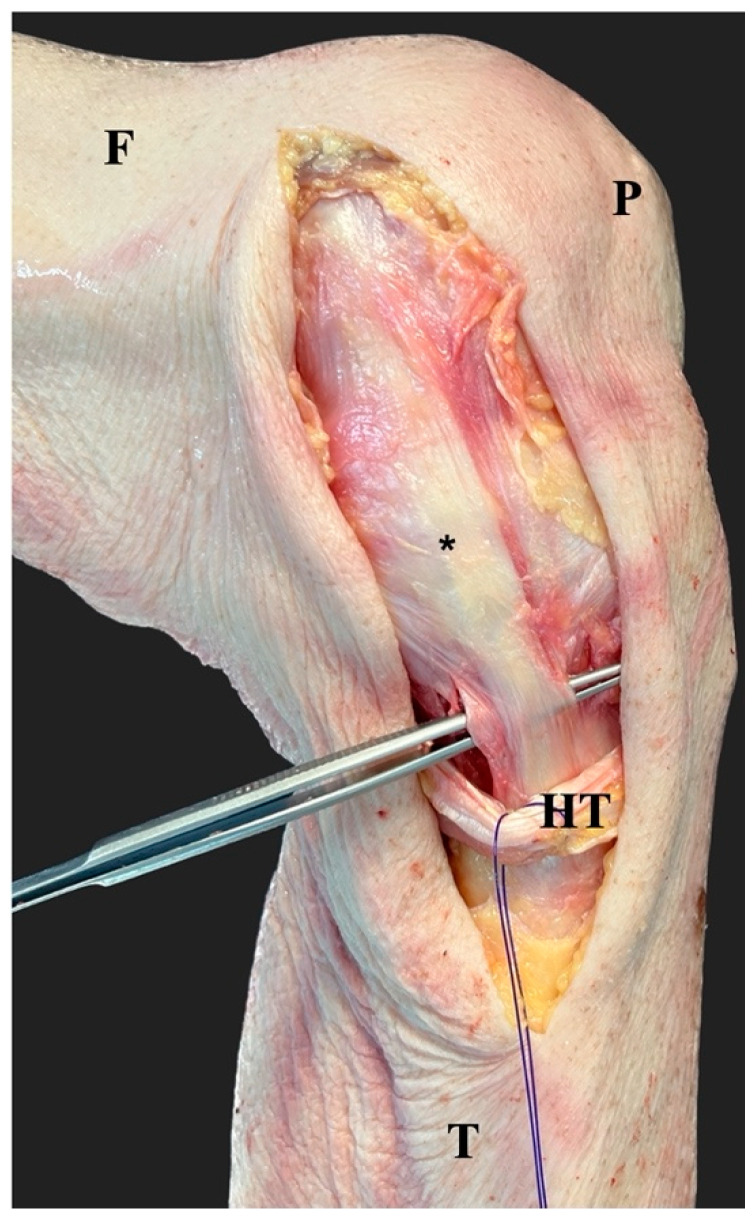
After the incision of the skin and the subcutaneous tissue, the fascia was incised in line with the skin incision. The hamstring tendons (HT) were preserved and the superficial medial collateral ligament (*) was identified. The forceps indicates the most distal part of the superficial medial collateral ligament. F, femur; T, tibia; P, patella.

Knees were stored at −24 °C and thawed at room temperature for 24 h prior to dissection. An anteromedial approach [14,16] was established via incision of the skin and subcutaneous tissue from the medial femoral condyle to the anteromedial aspect of the proximal tibia with a length of about 15 cm. Then, the anteromedial fascia was incised in line with the skin incision, and the hamstring tendons were preserved (Figure 1). The anteromedial retinaculum was sharply separated and mobilized from the anterior boarder of the superficial medial collateral ligament to expose the joint capsule [17]. For the extension of the anteromedial approach, the femoral and tibial insertions of the superficial medial collateral ligament and the posterior oblique ligament were sharply dissected and identified.

### 2.1. Osteotomy of the Distal Insertion of the Superficial Medial Collateral Ligament and Medial Epicondyle 

**Figure 2:**
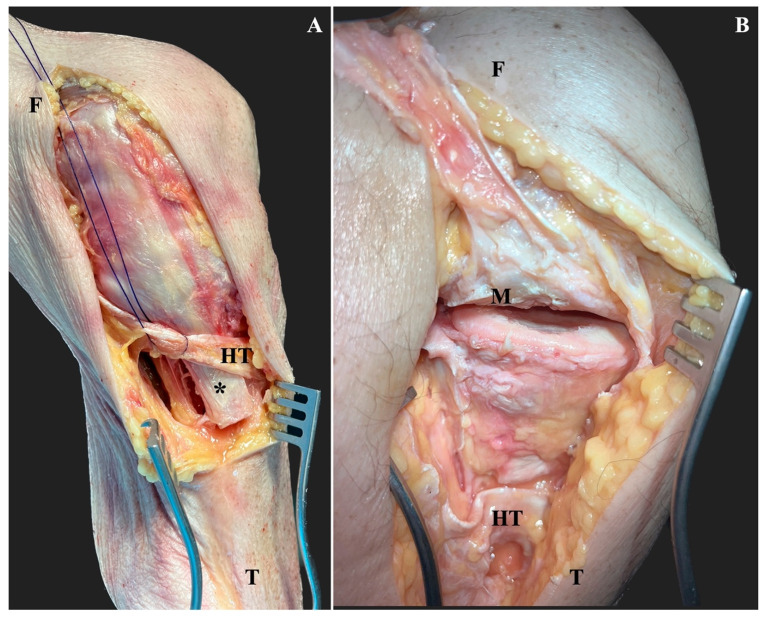
After identifying the superficial medial collateral ligament insertion underneath the hamstring tendons (HT), the tibial insertion osteotomized to a bone block with a length of 3 cm, a width of 1.5 cm and a thickness of 1 cm (*) (**A**). Then, the superficial medial collateral ligament was elevated proximally, together with the sharply detached deep MCL and the medial meniscus (M). Thereafter, the submeniscal arthrotomy was extended anteriorly and posteriorly to visualize the medial articular surface (**B**). F, femur; T, tibia.

After the identification of the distal bony insertion of the superficial medial collateral ligament underneath the hamstring tendons, about 6–8 cm distal to the joint line [18], a tibial bone block with a length of 3 cm, a width of 1.5 cm and a thickness of 1 cm was created using a 12 mm osteotome. This size of the bone block was chosen to properly reduce and fix it afterwards and to account for the wide insertion area of the superficial medial collateral ligament [18]. Then, the bone block and the superficial medial collateral ligament were reflected proximally. About 1–1.5 cm below the joint line, the deep collateral ligament had to be detached using a scalpel blade. Thereafter, the entire medial collateral ligament complex could be elevated together with the medial meniscus to expose the medial articular surface (Figure 2). To better expose the anteromedial articular surface, the capsule was incised horizontally under the meniscus to extend the submeniscal arthrotomy anteriorly as previously described [14].

**Figure 3:**
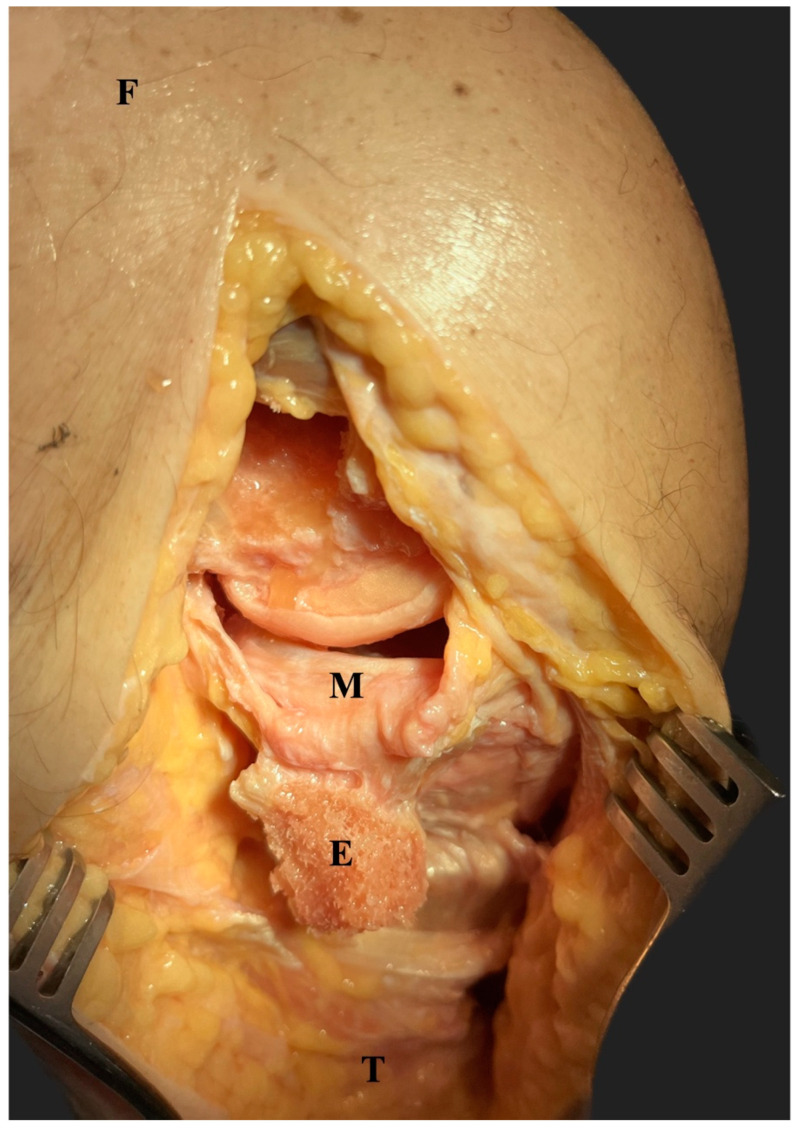
Using a 12 mm osteotome, a medial epicondylar osteotomy was performed (E). After distal deflection of the epicondyle, the medial meniscus became visible (M). F, femur; T; tibia.

Then, the tibial bone block was reduced and fixed with 2 3.5 mm cortical screws, and the medial femoral epicondylar osteotomy was performed as previously described [14]. Briefly, a bone block with a width and length of 4 cm and a thickness of 1 cm was created using a 12 mm osteotome. Care was taken to ensure that the insertions of both the superficial medial collateral ligament and the posterior oblique ligament were not damaged. The meniscus was left attached to the medial collateral ligament complex as exposure with sharp separation has been investigated previously (Figure 3) [14].

### 2.2. Measurement of the Exposed Medial Articular Surface

In the first three knees, the exposed medial articular surface was analyzed after the distal osteotomy, followed by the medial epicondyle osteotomy, and again after the distal osteotomy to rule out any measurement error due to the order of dissection. As these values did not differ from each other, the residual five knees were dissected in the order presented above. To quantify the exposed area of the medial tibial articular surface, a tactile 3-dimensional measuring arm (Absolute Arm 8320-7, Hexagon Metrology GmbH, Stockholm, Sweden) with an accuracy of ±0.05 mm was used. Using metrology software (PC-DMIS 2019 R1, Hexagon Metrology GmbH, Stockholm Sweden), a coordinate system was established by labeling the most prominent medial and lateral aspects of the tibial plateau proximally and the tibial shaft axis distally. Then, the visible articular surface of the medial tibial plateau was digitized with the tip of the measuring arm while applying valgus force to the knee. The digitized points were processed and projected to the coordinate system, and the area within the points could be calculated.

To relate the exposed area after medial epicondyle osteotomy and distal superficial medial collateral ligament osteotomy to the entire size of the articular surface of the medial tibial plateau, the knee was disarticulated and the medial articular surface area was digitized.

### 2.3. Statistical Analysis

For statistical analysis, IBM SPSS for MAC, version 29.0 (IBM Corp., Armonk, NY, USA), was used. The data are presented as absolute values and relative values with respect to the size of the entire medial tibial articular surface as percentages. The distribution of data was tested using the Shapiro–Wilk test. All data are presented as means and standard deviation. ANOVA with post hoc Bonferroni correction was used to analyze the differences in the visible articular surface area between the two extended medial approaches and the total medial articular surface. The significance level was set at *p* < 0.05.

## 3. Results

The mean size of the medial tibial articular surface was 12.7 ± 3.2 cm^2^ (range: 9.4–19.6). Following medial epicondyle osteotomy without submeniscal arthrotomy, 4.9 ± 1.0 cm^2^ (range: 3.7–6.7) of the anteromedial tibial articular surface was accessible. This corresponds to a relative area of 39.9 ± 9.7% (range: 26.8–53.2) of the entire medial articular surface. After distal superficial medial collateral ligament osteotomy, 9.7 ± 3.2 cm^2^ (range: 5.4–15.4) of the medial tibial articular surface was exposed. In relation to the size of the medial articular surface, the exposed area was 77.2 ± 16.9% (range: 44.5–99.0). While the measured area of the entire tibial plateau was not significantly different to the visible area following distal superficial medial collateral ligament osteotomy (*p* = 0.128), medial epicondyle osteotomy resulted in a significantly smaller exposed articular surface (*p* < 0.001). Comparing the size of the exposed medial articular surface after medial epicondyle osteotomy to the distal superficial collateral ligament osteotomy, the latter resulted in 37.3 ± 16.7% (range: 14.4–57.5) more exposure (*p* = 0.004). In contrast to the medial epicondyle osteotomy, the distal superficial medial collateral ligament osteotomy allowed for better visualization of the posteromedial and partially medial aspects of the medial tibial plateau (Figure 2).

## 4. Discussion

The most important finding of this study is that by extending an anteromedial approach to the tibial plateau with a distal superficial collateral ligament osteotomy, the medial articular surface exposure is superior to that using a medial epicondyle osteotomy without submeniscal arthrotomy. Additionally, due to the anatomy of the medial-sided soft tissues, when performing a distal superficial collateral ligament osteotomy, the medial meniscus does not need to be detached from the medial collateral ligament complex. Therefore, a distal osteotomy combines the advantages of superior joint exposure with less soft tissue damage and preservation of the vascularity of the medial meniscus compared to the standard medial epicondyle osteotomy.

Tibial plateau fractures are associated with a seven-times higher risk for osteoarthritis compared to the non-injured population [19]. Besides the damaged cartilage, the reasons for this increased risk may also be due to non-anatomic fracture reduction. It has been shown that residual intra-articular steps of more than 2.0 mm result not only in inferior patient-reported outcomes, but especially in elevated tibiofemoral contact forces, which over time, may result in early-onset osteoarthritis [10,11,12]. Thus, anatomic reduction should be the primary goal when treating subjects with tibial plateau fractures. However, intraoperative fluoroscopy does not sufficiently expose coronal plane fracture lines and intra-articular steps of less than 5 mm [13,20]. Thus, intraoperative computed tomography or direct visualization using adequate approaches are necessary to properly restore the articular surface. Additionally, not only can visualization be achieved using a surgical approach, but so can reduction and the placement of implants. If these medial-sided coronal plane fractures are not addressed with a proper buttress plate, the osteosynthesis is likely to fail, as has been shown clinically and biomechanically [21,22,23].

One may argue that medial sided impressions or comminution zones in tibial plateau fractures are rare. Nevertheless, the current literature indicates that in 8–10% of tibial plateau fractures have such a medial articular surface injury [7,8]. Frequently, this specific fracture pattern is due to an extension/hyperextension varus injury, resulting in a reduced medial proximal tibia angle and an anteromedial impression fracture [4]. As most of the medial side is covered by the wide superficial medial collateral ligament, only the anteromedial part of the articular surface can be seen using an anteromedial approach [14]. Extension of the anteromedial approach with a medial epicondyle osteotomy has been shown to increase the visible articular surface to almost the anteromedial two thirds of the articular surface [14]. This is only achieved if the medial meniscus is detached from the medial collateral ligament complex and retracted proximally. This, however, may result in deteriorated vascularization of the meniscus with subsequent issues regarding medial meniscus healing, as the blood supply comes from branches of the medial geniculate artery right between the medial meniscus and the medial collateral ligament complex [24,25]. If the medial meniscus is left attached to the medial collateral ligament complex in a medial epicondyle osteotomy, most of the exposed articular surface is covered by the meniscus.

Thus, in the current study, an osteotomy of the tibial insertion of the superficial medial collateral ligament was analyzed. This osteotomy allows for an extension of the anteromedial approach with a submeniscal arthrotomy without compromising the meniscocapuslar junction. By taking this approach, 77.2% of the entire medial articular surface was exposed and accessible, which was 37.3% more compared to a medial epicondyle osteotomy without submeniscal arthrotomy (*p* = 0.004). Krause et al. demonstrated that using a medial epicondyle osteotomy and submeniscal arthrotomy, 62.3% of the anteromedial tibial plateau is visualized. This is remarkably higher compared to the current study. The reason for this difference is the fact that in the current study, no submeniscal arthrotomy was performed in order to preserve the meniscocapsular junction. Thus, parts of the visible joint surface were covered by the medial meniscus. Compared to the distal osteotomy of the superficial collateral ligament with submeniscal arthrotomy, the findings of Krause et al. are similar to those of the current study [14]. Nevertheless, it seems that with a distal osteotomy, slightly more of the articular surface is exposed (about 15%).

Therefore, based on the current data, in the rare case of a necessary extension of an anteromedial approach, a distal osteotomy of the superficial medial collateral ligament may better expose the medial articular surface by preserving the blood supply to the medial meniscus. However, it is worth mentioning that the posteromedial- and medial-most aspects of the medial tibial articular surface may not be visible even using extended approaches [14]. The reduction and fixation of the distal superficial collateral ligament osteotomy can be performed with slight flexion and using 2 3.5 mm bicortical screws. When opting for a distal osteotomy of the superficial medial collateral ligament, surgeons must make sure that the fracture patterns allows for such an osteotomy, as in fractures extending towards the insertion of the ligament, fixation of the fragment might be challenging. Additionally, especially in fracture dislocations, the integrity of the medial collateral ligament should be checked before performing the osteotomy.

The limitations of this study include the fact that the surgical approaches and measurements were performed on cadaveric specimens. Intraoperatively, the articular surface exposure may be much less as the surrounding soft tissues and muscles may not be as loose as in cadaveric specimens. Further, the number of specimens was relatively low. Additionally, when performing the measurements, knees were placed at various degrees of flexion via manual valgus stress. The amount of valgus stress, however, was not standardized. However, this is the best reflection of daily clinical practice. Additionally, a submeniscal arthrotomy was not analyzed for the medial epicondyle osteotomy. This was for two reasons. First of all, this has been described and published previously [14], and secondly, it cannot be expected that the results of the exposed articular surface will be different between the proximal and distal osteotomy when a submeniscal arthrotomy is performed. The main advantage of a distal osteotomy of the superficial collateral ligament, however, is that the meniscocapsular junction, and therefore, the blood supply to the medial meniscus is preserved.

Clinical studies are needed to prove the applicability of this new surgical approach.

## 5. Conclusions

The data from this study suggest that a distal superficial collateral ligament osteotomy may be superior to a medial epicondyle osteotomy when an extension of the anteromedial approach to the tibial plateau is required. A distal superficial medial collateral ligament osteotomy combines the advantages of better exposure of the medial articular surface with preservation of the blood supply to the medial meniscus. However, surgeons should carefully consider whether such an extended approach is necessary, as it significantly increases invasiveness.

## Data Availability

The data can be requested from the authors.
